# Larval Rearing Temperature Influences Amount and Composition of the Marking Pheromone of the Male Beewolf, *Philanthus triangulum*

**DOI:** 10.1673/031.010.7401

**Published:** 2010-06-25

**Authors:** Kerstin Roeser-Mueller, Erhard Strohm, Martin Kaltenpoth

**Affiliations:** ^1^ University of Regensburg, Department for Zoology, 93040 Regensburg, Germany; ^2^ University of Würzburg, Department of Animal Ecology & Tropical Biology, 97074 Würzburg, Germany; ^3^ Present address: Max Planck Institute for Chemical Ecology, Research Group Insect Symbiosis, Hans-Knöll Strasse 8, 07745 Jena, Germany

**Keywords:** development time, life history traits, rearing temperature, sex pheromone, survival, weight

## Abstract

Pheromones play an important role for courtship and mating in many insect species, and they are shaped by a complex interaction of genetic and environmental factors. Developmental temperature is known to have a strong influence on adult life history, morphology, and physiology, but little is known about its effect on pheromone characteristics. In the present study, the influence of temperature during larval development on the amount and composition of the complex marking pheromone from the cephalic glands of the adult male beewolf, *Philanthus triangulum* F. (Hymenoptera: Crabronidae), was investigated. Additionally, the effects of temperature on several life-history traits were examined. European beewolf larvae were reared at three constant temperatures (20, 25, and 30° C). Males reared at 20° C showed longer development times and higher mortality, suggesting that low temperatures constitute stressful conditions for developing larvae. After eclosion, the amount and composition of the scent marking secretion of the adult males was analyzed by coupled gas chromatography-mass spectrometry. Males that had been reared at 20° C had significantly less secretion than individuals reared under warmer conditions (25° C and 30° C). Furthermore, larval rearing temperature had a significant effect on the composition of the adult males' pheromone gland content, with warmer rearing conditions leading to higher relative amounts of compounds with high molecular weight. The results show that the temperature during larval development significantly affected the amount and composition of the content of the male pheromone glands, probably due to physiological constraints and competing processes for limited energetic resources. Thus, the pheromone gland content may contain information on developmental conditions of males, which may have consequences for female mate choice decisions and male reproductive success.

## Introduction

In many organisms, communication between mating partners is mediated by pheromones (e.g. Jones and Hamilton 1998; Johansson and Jones 2007). These chemical signals do not only facilitate the localization and identification of conspecific individuals (Johansson and Jones 2007), they may also contain information on the morphological and/or physiological condition of the potential mating partner, e.g. size, age, symmetry, fertility, or immunocompetence (Jones et al. 1998; Marco et al. 1998; Martín and López 2000; Reusch et al. 2001). This information may provide a potential partner with cues for an adaptive mate choice. Mating with a highquality partner might provide direct (e.g. reduced risk of parasite infection, higher chances of receiving fertile gametes, defense, brood care) and/or indirect benefits (e.g. “good genes” for the offspring) to choosy individuals (Eisner and Meinwald 1995; Andersson and Iwasa 1996; Penn and Potts 1998; Wagner and Harper 2003).

For a number of species, a genetic basis for the variation in pheromone characteristics has been shown (Collins and Cardé 1985; Sappington and Taylor 1990a; Roelofs and Rooney 2003; Herzner et al. 2006; Sheck et al. 2006). Some authors also found that the amount and/or composition of sex pheromones was influenced by environmental factors (Sappington and Taylor 1990b; Clark et al. 1997; Moore 1997). However, most of these studies investigated the effect of environmental conditions on pheromone characteristics during the adult stage. The impact of conditions during larval development has received little attention (see e.g. Conner et al. 1990; Ono 1993; Tillman et al. 1999).

Temperature is one of the most important environmental factors since it influences many morphological, physiological, and life-history traits, e.g. size, fecundity, and development time (Ratte 1984; Atkinson 1994; Blanckenhorn 1997; Nabeta et al. 2005). In some adult Lepidoptera, ambient temperature has an immediate impact on pheromone amount and/or composition probably by directly affecting biochemical pathways (Ono 1994; Raina 2003). In potato tuberworm moths, different rearing temperatures during larval development resulted in changes in the amount and composition of the females' sex pheromone (Ono 1993).

The effect of environmental conditions on pheromone composition may reduce the efficiency of communication processes or even result in the loss of signal function. Thus, a certain degree of developmental stability has to be expected to retain the signal's information content for conspecifics (Paterson 1985; Møller and Swaddle 1997). On the other hand, variation in pheromone composition due to environmental effects might also increase the information content of a signal. Assuming that a male pheromone varies in response to different environmental conditions in a defined way and that these changes have some fitness implications, a female might be able to assess some indicator for quality from pheromone composition. For example, if the temperature during development depends on decisions and/or competitive abilities of a male's mother (e.g. by choosing and defending suitable oviposition or nesting sites), a male that developed at optimal temperatures might be more attractive to females. If these maternal characteristics are heritable, choosing a son of such a female as a mating partner should be beneficial for a female (Andersson 1994; Møller and Alatalo 1999). Here, one precondition for this scenario was investigated: this study was designed to determine whether developmental temperatures affect quantity and composition of the male pheromone of a hymenopteran species, the European beewolf, *Philanthus triangulum* F. (Hymenoptera: Crabronidae).

*P. triangulum* are solitary digger wasps that live in warm and sandy areas (Strohm and Linsenmair 1995). Females excavate a nest burrow with several separate brood cells, each of which is provisioned with one to five paralysed honeybees (*Apis mellifera*) as food for the developing larva (Strohm and Linsenmair 1995, 1997, 2000, 2001). To prevent fungal infestation in the warm and humid brood cell, the prey is preserved by the females by embalmment with a gland secretion (Herzner and Strohm 2007). Moreover, *P. triangulum* females provide the brood cell with a whitish substance that originates from specialized antennal glands (Göttler et al. 2007) and that contains symbiotic bacteria that protect the cocoon from fungal infestation (Kaltenpoth et al. 2005; Kaltenpoth et al. 2006).

*P. triangulum* males establish and defend small territories (about 0.25 m^2^) in the vicinity of female nest aggregations (Simon-Thomas and Poorter 1972; Strohm 1995). They scentmark these territories with a pheromone from cephalic glands to attract receptive females (Simon-Thomas and Poorter 1972; Evans and O'Neill 1988; Strohm 1995; Strohm and Lechner 2000; Schmitt et al. 2003). Territories of different males are often aggregated, thereby forming a lek that might facilitate female choice on the basis of the competing males' pheromone quality and quantity (Simon-Thomas and Poorter 1972; Evans and O'Neill 1988; Kroiss et al. 2010). Females approach these territories from the downwind side in a zig-zagging flight pattern, probably orienting toward the windborne pheromone (Evans and O'Neill 1988). Since the copulation is not preceded by any kind of visual display, female choice appears to be based, at least predominately, on information obtained from the male's secretion (E. Strohm and M. Kaltenpoth, unpublished observation). Although the pheromone components are rather long chained and, thus, intuitively seem to not be volatile, there is ample evidence that most of the compounds are volatile enough to be detected by olfaction (Herzner et al. 2005; Schmitt et al. 2007) without contact. There is no conspicuous antennation of the territory by females, and there is no visual courtship by males. Females simply alight in a territory, the male approaches her, sits on her back and inserts its genitalia. The scent marking secretion consists of at least 55 components, including long-chain aliphatic hydrocarbons and some compounds with functional groups (Schmitt et al. 2003; Kroiss et al. 2006). These components are also found in extracts from male territories (E. Strohm, T. Schmitt, G. Herzner, J. Kroiss and M. Kaltenpoth, unpublished data). It is already known, that the amount and composition of the male marking pheromone is influenced by family affiliation (Herzner et al. 2006; Kaltenpoth et al. 2007), age (Kaltenpoth and Strohm 2006), and geographical origin (Kaltenpoth et al. 2007).

Male *P. triangulum* were reared at different temperatures, and mature male pheromone quantity and composition was analysed using coupled gas chromatography and mass spectrometry. Several life-history traits (survival probability of the larva, development time, and adult weight) were chronicled. Moreover, it was assessed whether there were predictable trends in the temperature-dependent variation of the pheromone composition regarding the chain length of the individual pheromone substances. The results are discussed with regard to possible physiological and ecological constraints and their relevance for mate choice.

## Materials and Methods

### Specimens and rearing conditions

Female *P. triangulum* were taken from a laboratory population that represented the F1 generation of females caught from populations in the vicinity of Würzburg, Germany. They were kept individually in observation cages that consisted of a flight compartment and an attached nesting area where the females could establish their nests (see Strohm and Linsenmair 1995). The cages were kept in a greenhouse at 25 ± 5° C, with average humidity at 45% (range: 30–80%) and additional illumination of the flight cage by neon lamps for 14 h per day. The females were provided with honey and live honeybees *ad libitum*, and each cage was checked several times each day for new brood cells. The content of brood cells with two bees was transferred to artificial brood cells in Petri dishes: each Petri dish was filled with a fixed amount (130 g) of autoclaved sand, and an artificial, cylindrical brood cell with a standardized diameter of about 2.6 cm and a depth of 1 cm was formed. The paralyzed bees with the *P. triangulum* egg, as well as the white antennal gland secretion containing the symbiotic bacteria (Kaltenpoth et al. 2005; Kaltenpoth et al. 2006) were transferred to the artificial brood cell. The humidity in the brood cell was kept constant at 4% H2O (w/w) by weighing brood cells every other day and replacing evaporated water through four small holes in the lid of the Petri dish.

Each brood cell was randomly assigned to one temperature treatment. Experimental brood cells were stored in three conditioning cabinets (ATS1373 So, Ehret GmbH) at 20, 25, or 30° C. The eggs and larvae were examined every day, and life-history parameters were recorded (hatching date, cocoon spinning, eclosion from the cocoon, occurrence of mould infestations, and death of the larva).

Each emerged adult male was weighed (Mettler AE 160; ± 0.1g), individually marked on the thorax with dots of acrylic paint, released into a climate chamber (2.4 m × 1.8 m × 2.1 m, 25/20° C day/night and 12:12 L:D) containing sand-filled buckets for digging sleeping burrows, and provided with honey *ad libitum.* Since *P. triangulum* males need about 5–9 days to develop the complete pheromone blend (Kaltenpoth and Strohm 2006), they were left in the climate chamber until the age of 12 days. Then, they were caught, transferred into small polystyrol vials (height: 80 mm, diameter: 35 mm), and provided with moist sand and honey for another two days. During this time, males that had depleted their pheromone in the climate chamber by scent marking could replenish their pheromone glands. Finally, the males were anesthetized with CO_2_, killed by freezing, and kept at -30° C until chemical analysis.

### Chemical analysis

The frozen males were thawed and decapitated, and their heads were incised on both sides below the eyes to open up the postpharyngeal gland, which is the storage organ of the male sex pheromone (Kroiss et al. 2006; Herzner et al. 2007). Heads were placed individually in glass vials (1.5 ml), and 20 µl of a solution of 1 µg/µl octadecane in hexane (equivalent to a final amount of 20 µg of octadecane) was added as an internal standard to each vial to allow absolute quantification of the pheromone. The heads were then submerged in approximately 1 ml of distilled hexane, and the gland contents were extracted for four hours. The extracts were immediately analyzed by coupled capillary gas chromatography-mass spectrometry with an Agilent 6890N Series gas Chromatograph (Agilent Technologies, www.agilent.com) coupled to an Agilent 5973 mass selective detector. The gas Chromatograph was equipped with a DB-5ms + fused silica capillary column (J & W (Agilent), 30 m × 0.25 mm ID; df = 0.25 µm; temperature program: from 60° C to 300° C at 5° C/min, held constant for 1 min at 60° C and for 10 min at 300° C). Helium was used as the carrier gas with a constant flow of 1 ml/min. A split/splitless injector (250° C) was used with the purge valve opened after 60 sec. The electron impact mass spectra were recorded with an ionization voltage of 70 eV, a source temperature of 230° C, and an interface temperature of 315° C. Since preliminary analyses had revealed that the total amount of chemicals in the sample has an effect on the detection and quantification of certain components, samples in which the concentration of the gland extract was either too high (overlapping peaks) or too low (with compounds below detection threshold) were rerun after adjusting the pheromone concentration by addition or evaporation of hexane.

In the pheromone gland extracts, 21 components could be reliably detected in all samples, and their peaks were manually integrated with MSD ChemStation software (Agilent). The substances were identified by comparison of mass spectra and retention times with earlier analyses (Schmitt et al. 2003; Kroiss et al. 2006). Not all substances described as components of the pheromone by Kroiss et al. (2006) could be detected due to the low concentrations of the gland content extracted from single males. The peaks of (Z)-11-eicosen-1-ol and (Z)-9-tricosene were not fully separated in all chromatograms and were therefore pooled and treated as one peak for the statistical analyses. Because of the much higher eicosenol fraction (Schmitt et al. 2003) the peak is labelled as “eicosenol” in the following. This procedure is conservative with regard to the hypotheses tested. For each sample, the total peak area was standardized to 100%, and the relative amount of each peak was calculated. Since the relative peak areas represent compositional data, they were transformed to logcontrasts prior to statistical analyses (Aitchison 1986). Using the octadecane peak as an internal standard, the total amount of the gland content was estimated.

### Statistical analysis

The influence of the rearing temperature on development time and adult weight was analyzed by Kruskal-Wallis tests, χ^2^-tests were used to evaluate the survival rate, and analyses of variance and covariance (with adult weight as the covariate) were conducted to assess the influence of temperature on the quantity of the pheromone gland content (including post hoc tests with Scheffe's multiple comparisons). Tests were calculated using BIAS 8.1 and SPSS 11.0.

Multivariate analyses were conducted to test for differences in the chemical profiles of males reared at different temperatures: The peaks were subjected to a principal component analysis (PCA with varimax rotation, principal components with eigenvalues > 1 were included in the subsequent analyses) to reduce the number of describing variables. The extracted principal component analysis factors were then subjected to a discriminant analysis to assess whether males confronted with different rearing temperatures exhibit differences in their pheromone profiles. To investigate the influence of the rearing temperature on individual components of the pheromone gland, a multivariate analysis of variance (MANOVA) was conducted. Additionally, a correlation analysis was used to test for an influence of temperature on the relative amounts of the substances depending on molecule size (either chain lengths or molecular weights). SPSS 11.0 was used for the calculation of all tests.

Earlier studies revealed a distinct chemical dimorphism in the pheromone blend of *P. triangulum* males (Kroiss et al. 2006), with either Z-(9)-pentacosene (C_25_-type) or Z-(9)-heptacosene (C_25_/C_27_-type) as the major component. Therefore, all analyses on the composition of the pheromone gland extracts were conducted with the data of the C_25_-chemotype only, in addition to the analysis including both chemotypes. The sample size of C_25_/C_27_-type individuals was too small to allow separate analyses. Since the analyses including both types and those including only C_25_-type males yielded qualitatively identical results, only the data based on the complete dataset are presented here.

## Results

### Life history traits

Development time of males differed significantly between temperature groups (Kruskal-Wallis test, H = 39.7, df = 2, p < 0.001; Figure 1). On average, males reared at 30° C needed 20 days from the egg stage until eclosion from the cocoon, whereas males reared at 20° C had just started cocoon spinning at that point in time (Q_10_ value of average development times = 3.33). Male adult weight was not significantly affected by rearing temperature (Kruskal-Wallis test, H = 0.441, df = 2, p = 0.802). Survival probability of developing males, however, was strongly influenced by rearing temperature (contingency test, χ^2^ = 9.24, p = 0.01). Males reared at 20° C had a significantly higher mortality rate (74%) than males in any of the other two temperature groups (25° C: 39% mortality, 30° C: 43% mortality; pairwise comparisons: 20° C versus 25° C: χ^2^ = 7.435, p = 0.006; 20° C versus 30° C: χ^2^ = 4.795, p = 0.029, 25° C versus 30° C: χ^2^ = 0.015, p = 0.904).

### Quantity of the pheromone gland content

**Figure 2.**
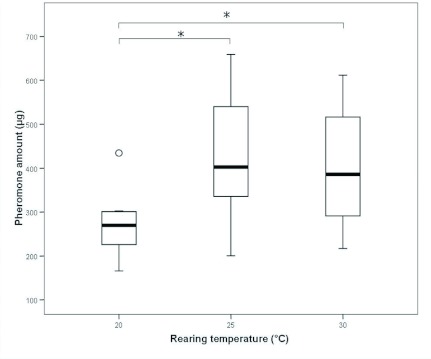
Absolute marking pheromone amount (in µg) of adult *Philanthus triangulum* males reared at different temperatures (N_20° C_ = 7, N_25° C_ = 22, N_30° C_ = 14; * significant at p < 0.05). Bold lines represent medians, boxes comprise the interquartile range, and whiskers indicate minimum and maximum values, except outliers, which are represented by circles. High quality figures are available online.

The total amount of the content of the pheromone gland from male heads ranged from 165 to 659 µg (mean ± SD = 387 ± 125 µg). The temperature during larval development had a significant influence on the quantity of marking pheromone of adult males (ANOVA, F_2,40_ = 4.86, p = 0.013, *n* = 43). Since body weight might be a confounding factor for the amount of gland content (Pearson's regression, regression coefficient r = 0.354; p = 0.020), an analysis of covariance was conducted with body weight as covariate, but the temperature effect persisted (ANCOVA; F_2,39_ = 4.189, p = 0.022, *n* = 43): Adult males reared at 20° C had significantly less marking pheromone than males reared at either 25° C or 30° C (Scheffe's multiple comparisons, p < 0.05; Figure 2).

### Composition of the pheromone gland content

**Figure 3.**
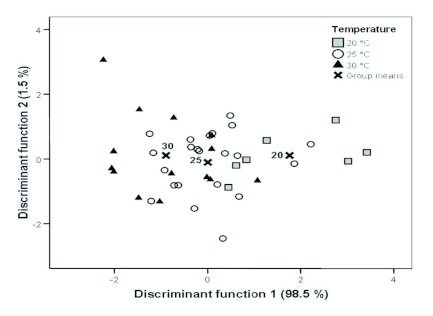
Discriminant analysis of the marking pheromone composition of males with different rearing temperatures (squares: 20° C, *n* = 7; circles: 25° C, *n* = 22; triangles: 30° C, *n* = 14). High quality figures are available online.

In the extracts of male *P. triangulum* heads, 21 compounds were found in all samples and were included in the analysis (Table 1), with (Z)-11-eicosen-1-ol (including minor amounts of (Z)-9-tricosene) constituting the component with by far the highest relative amount (mean ± SD = 61.21 ± 3.83 %). The principal component analysis produced five principal components with eigenvalues larger than 1 (eigenvalues: 5.8, 3.8, 3.5, 2.1, 1.5), explaining 79.9% of the total variance. The discriminant analysis on these principal components significantly separated the three temperature groups (Wilks λ = 0.541, χ^2^ = 24.3, df = 4, p < 0.001, *n* = 43; Figure 3). Between 57 and 64% (average = 60.5%) of the males were correctly assigned to the temperature treatment by the discriminant analysis. Only 33% of correct classifications would be expected by chance.

**Figure I.**
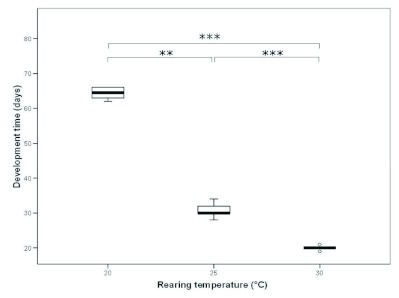
Median development time (oviposition to eclosion of imago) of *Philanthus triangulum* males at different rearing temperatures (N_20° C_ = 7, N_25° C_ = 24, N_30° C_ = 17; ** significant at p < 0.01, *** significant at p < 0.001). Bold lines represent medians, boxes comprise the interquartile range, and whiskers indicate minimum and maximum values, except outliers, which are represented by circles. High quality figures are available online.

**Table 1.**
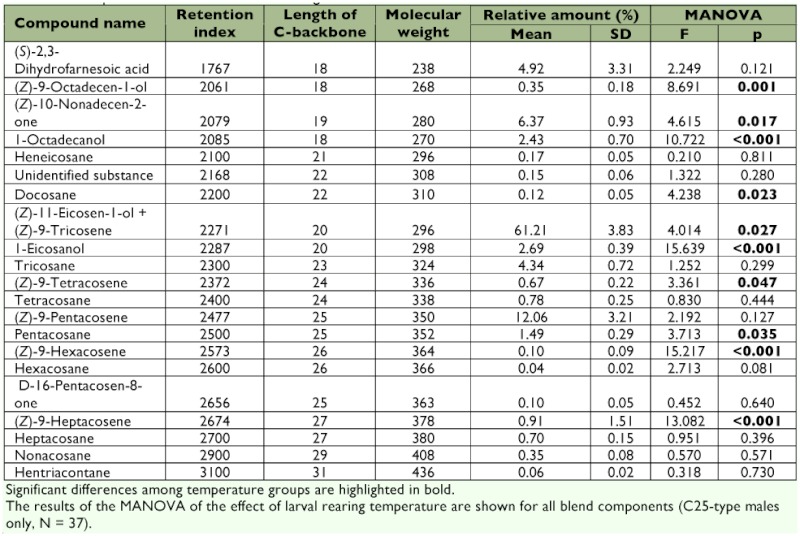
Compounds of the males' scent marking secretion.

A multivariate analysis of variance (MANOVA) was conducted with the 21 components of the pheromone gland extracts to elucidate the contribution of the individual components of the pheromone blend to the differences between rearing temperatures (Table 1). Since several components differ significantly in their relative amount between the two chemotypes (Kroiss et al. 2006), only males of the C_25_-type (*n* = 37) were included in this analysis. The MANOVA revealed a significant temperature effect on the composition of the marking pheromone (p = 0.035, Pillai's trace = 1.453, F = 1.896). Ten of the 21 components (five with and five without functional groups) differed significantly in their relative amounts among temperature groups (Table 1).

**Figure 4.**
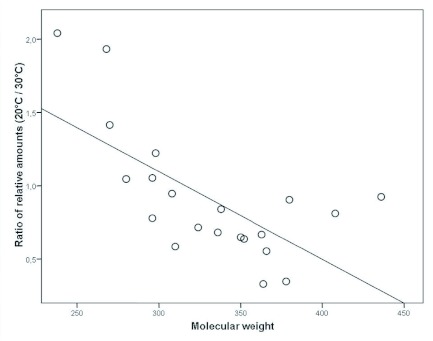
Correlation between the molecular weight of each component and the ratio of its mean relative amounts at 20° C and 30° C High quality figures are available online.

To test whether the temperature dependent changes in pheromone composition show a trend with regard to the size of the molecules, a correlation analysis of the molecular weight of each component and the ratio of its relative amounts at 20 and 30° C was conducted (Figure 4). The analysis showed a significant negative correlation (Pearson correlation, correlation coefficient r = -0.67; p = 0.001). Thus, males reared at a low temperature (20° C) exhibited significantly higher relative amounts of components with low molecular weights, while the blend of males reared at a high temperature (30° C) contained higher amounts of the hydrocarbons with higher molecular weights. The same effect was found if the hydrocarbon chain length (number of C atoms) was used instead of the molecular weights (Pearson correlation, correlation coefficient r = -0.72; p < 0.001).

## Discussion

### Life history traits

Larvae reared under higher ambient temperatures exhibited significantly shorter development times and lower mortalities than those kept under low-temperature conditions. Such negative correlations between temperature and development time have been described for numerous insect species (Ratte 1984) and have also been found in earlier studies on *P. triangulum* (Strohm 2000). This effect is generally ascribed to the temperature-dependence of basal biochemical processes (Schmidt-Nielsen 1999). The temperature tolerance of an organism follows an optimum curve (Ratte 1984; Schmidt-Nielsen 1999), and deviations from the ideal temperature range lead to developmental stress or can be lethal. Generally, a short development time is advantageous, because it might reduce the mortality risk in the vulnerable larval stage (Sibly and Calow 1986). Selection for a fast development is likely to be particularly strong in *P. triangulum*, because the larvae are exposed to a high density of pathogenic microorganisms in their subterranean brood cells and, thus, face a high risk of bacterial or fungal infestation (Strohm and Linsenmair 1998, 2001; Kaltenpoth et al. 2005; Herzner and Strohm 2007). *P. triangulum* females in Central Europe strongly prefer areas with favourable climatic conditions (i.e. warm places with high solar irradiation) for nesting (Olberg 1953; Rathmayer 1962) and this is likely to reduce larval development times and enhance the survival probability of the offspring. Additionally, short larval development times may allow the emergence of a second generation in one flight season.

### Amount of pheromone gland content

The results suggest that low larval rearing temperatures cause a reduction in the quantity of pheromone gland contents of adult *P. triangulum* males. Males reared at 20° C possessed significantly less marking pheromone than those reared at 25 and 30° C, and this effect was not caused by differences in body size. Suboptimal developmental conditions probably lead to competing processes for limited energetic resources causing a higher investment into traits important for current survival, such as immunocompetence, at the expense of future reproductive traits (Ratte 1984; Zwaan et al. 1992; Landete-Castillejos et al. 2002; Buchanan et al. 2003; Kemp and Rutowski 2007). For example, the number of pheromone gland cells (Göttler and Strohm 2008) could be reduced when males are confronted with developmental stress, resulting in reduced pheromone production.

In *P. triangulum*, the characteristics of the male sex pheromone and gland morphology suggest that pheromone quantity probably is a crucial factor for male reproductive success. First, regarding the huge amount of gland content applied to the territory, the pheromone constitutes an exaggerated signal targeted at the females' sensory sensitivity (Herzner et al. 2005). The conspicuousness of a male territory for females is probably positively correlated with the amount of secretion applied to the territory. Thus, sexual selection may promote a high rate of pheromone production. Second, the gland tissues involved in the production and the storage of the pheromone are greatly enlarged, and details of their morphology suggest a high level of metabolic activity (Göttler and Strohm 2008). There is evidence that territory owners deplete most of their marking pheromone over their daily activity period and that they replenish the stores overnight (E. Strohm, unpublished data). Thus, the amount of gland content available to a male probably limits its ability to scent mark its territory and to attract receptive females. Most substances applied to the substrate are quite long-lasting (J. Kroiss, unpublished data), but independent of the degree of volatilization of different components, males that are able to produce larger amounts of pheromone within a given time probably have a selective advantage because the more pheromone that is applied the more conspicuous or attractive will it be for females. Correlations between pheromone production and male reproductive success have been shown for several other insect taxa (e.g. Löfstedt et al. 1990; Droney and Hock 1998).

### Composition of the pheromone gland content

There was a significant influence of the larval rearing temperature on the composition of the pheromone gland content of adult male *P. triangulum*. These changes in composition were gradual, and there was no loss or addition of components. Although there was a broad overlap between the blends of males reared at 20, 25, and 30° C, the three groups were significantly separated by a discriminant analysis (Figure 3). The relative amounts of about half of the components of the marking pheromone differed significantly among groups (Table 1). Interestingly, changes in the relative amounts of the components with the rearing temperature were correlated with the molecule size of the substances with higher temperatures leading to an increase in the proportions of long chained components (Figure 4). Thus, there is a predictable trend in the temperature dependent variation of the composition of the marking pheromone.

Changes in the ratio of pheromone components due to different larval rearing temperatures have been described for the moth *Phtorimaea operculella* (Lepidoptera: Gelechiidae; Ono 1993). This effect has been ascribed to physiological limitations and different temperature dependencies of enzymes involved In contrast to the *P. triangulum*, however, in *P. operculella*, the females are the pheromone-producing sex, as is the case in most moth species (Svensson 1996).

Since sexual signals produced by males and females are subjected to considerably different selection pressures (Phelan 1992, 1997), the fitness consequences of temperature-induced changes in pheromone composition are expected to differ between the sexes as well. Due to asymmetric parental investment (Andersson 1994), females should be more discriminating than males when choosing a mating partner. The influence of larval conditions on the composition of the male sex pheromone potentially provides information for female choice. There are several possibilities why it might be beneficial for a female to choose a male with regard to its rearing temperatures. First, a male that developed at an optimal temperature might be healthier and more fertile than others, thereby potentially providing direct benefits, such as more viable sperm, to females. Second, assuming that ideal nesting sites are limited (Strohm et al. 2001) only competitively superior females might be able to defend such sites. Thus, choosing a male whose pheromone indicates optimal temperatures during development might be beneficial for a female since this male may carry high quality alleles that it inherited from its mother.

The results of this study show that the temperature during larval development has a significant effect on the amount and composition of the marking pheromone of adult male *P. triangulum.* These changes may reflect physiological constraints and competing processes for limited energetic resources during early development. To the authors' knowledge, this is the first study on the effects of developmental temperature on amount and composition of the pheromone gland content in a species with a male sex pheromone. The enormous importance and widespread distribution of chemical signals as well as the potentially high information content of male sex pheromones and its implications for female mate choice decisions call for further studies on the impact of developmental conditions on pheromone characteristics.
